# The Cost of Regulating Effort: Reward and Difficulty Cues With Longer Prediction Horizons Have a Stronger Impact on Performance

**DOI:** 10.5334/joc.415

**Published:** 2025-01-07

**Authors:** Nanne Kukkonen, Senne Braem, Jens Allaert, Joshua O. Eayrs, Nicoleta Prutean, S. Tabitha Steendam, C. Nico Boehler, Jan R. Wiersema, Wim Notebaert, Ruth M. Krebs

**Affiliations:** 1 Department of Experimental Psychology, Ghent University, Belgium; 2 Department of Head and Skin, Ghent University, Belgium; 3 Department of Experimental Clinical and Health Psychology, Ghent University, Belgium

**Keywords:** Cognitive effort, effort regulation, decision making, cognitive control, random-dot-motion, Stroop

## Abstract

Many theories on cognitive effort start from the assumption that cognitive effort can be expended at will, and flexibly up- or down-regulated depending on expected task demand and rewards. However, while effort regulation has been investigated across a wide range of incentive conditions, few investigated the cost of effort regulation itself. Across four experiments, we studied the effects of reward expectancy and task difficulty on effort expenditure in a perceptual decision-making task (random-dot-motion) and a cognitive control task (colour-naming Stroop), and within each task comparted cues between short (cueing the next trial) and long (cueing the next six trials) prediction horizons. We found that participants used the cue information only when it was valid for multiple trials in a row. In the random-dot-motion task, a high reward expectancy resulted in better accuracy, especially in easy trials, but only with long prediction horizon. Similarly, in the Stroop task, the reward facilitation of reaction time was only observed after reward cues with a long prediction horizon. Together, our results indicate that people experience a cost to effort regulation, and that lower adjustment frequency can compensate for this cost.

## Introduction

One of the most robust findings in studies of human behaviour has been that incentives motivate effort allocation, and are close to a prerequisite for it (Inzlicht et al., 2014). A similarly robust finding has been that mental effort is experienced as aversive (for meta-analysis, see David et al., 2024), and is preferentially avoided (Kool et al., 2010; but see Clay et al., 2022 & Inzlicht et al., 2018), in accordance with the law of least effort (Hull, 1943). These two findings have led to the operational definition of mental effort as a decision-making process, in which the inherent cost of effort is offset by incentives. This definition has been formalised in neuroeconomic (Expected Value of Control; Shenhav et al., 2013) and neurocomputational (Verguts et al., 2015) models that account for the extent of effort regulation in tasks that orthogonally manipulated various levels of reward and difficulty (Krebs et al., 2012, Vassena et al., 2019) or the performance-contingency of reward (Frömer et al., 2021). Most models have thus focussed on this evaluative process, based on the assumption that effort is up- and down-regulated based on the current/expected reward and difficulty context. Here, we aim to critically evaluate this assumption, and investigate the possibility that this effort regulation might come with its own cost.

Considering effort regulation as a decision-making process by which humans engage in effortful behaviour in an optimal way, effort exertion should be dynamic and goal-directed, leading to improvements in performance whenever it is worth it. Motivated by this notion, previous studies have used various cueing paradigms where cues inform participants of the difficulty level of the upcoming task and/or the reward they can gain (usually for fast correct responses). In accordance with resource-rational models of effort, presentation of (high) reward cues has been shown to improve performance in tasks spanning from visual discrimination (Krebs et al., 2012; Schevernels et al., 2014) to conflict processing (Krebs et al., 2010; Padmala & Pessoa, 2011) and response inhibition (Boehler et al., 2014; for a recent meta-analysis see Burton et al., 2021). In addition to trial-wise incentive manipulations, some evidence exists for reward ameliorating attentional decrements in a sustained effort task (Esterman, et al., 2016; Esterman, et al., 2017). Despite these past investigations on effort exertion, none have systematically investigated whether prediction horizon of reward and difficulty cues (i.e. number of consecutive trials to which the cue applies) influences effort expenditure.

Although the inherent cost of effort is widely assumed to scale with objective task difficulty (Westbrook et al., 2013), it is unclear whether a cost also exists for regulating effort dynamically with changing effort-related cues. A long tradition of task switching research has shown that this is not a trivial operation, as undertaking one task after being engaged in another comes with a cost in performance (Allport et al., 1994; Braem & Egner, 2018; Monsell, 2003). Furthermore, this cost is subjectively experienced as aversive (Vermeylen et al., 2019), and participants tend to avoid it (Kool et al, 2010; Vermeylen et al., 2022). While a change in effort allocation (within a task) does not constitute a change in task settings, a shift in the overarching goal to invest more effort in a given task could still be considered a shift in an arguably more abstract action plan (e.g., Fine & Hayden, 2022). Keeping track of changing incentive and difficulty information in the environment necessitates dynamic adaptations in effort processing and can incur costs analogous to those in a task switching context (for a similar reasoning, see Grahek et al., 2024). Specifically, we hypothesized that when constantly presented with different effort and reward cues with a limited prediction horizon (e.g., only pertaining to the next trial), people might choose to forego consideration of the cues, in line with the idea that the constant regulation of effort is simply not worth the effort – or at least not valuable enough in face of the expected overall outcome.

In addition to a lack of research on the cost of dynamic effort regulation, the consequences of difficulty expectation in cognitively effortful tasks have been overlooked in effort literature, likely due to the design challenge it presents. While the motivational consequences of reward expectation can be tested by comparing the effects of no (or low) reward expectancy cues versus (high) reward expectancy cues on task performance, the effect of task difficulty anticipation of effort exertion has been routinely confounded by the actual difficulty of the task always corresponding to the cued demand. This has prevented inferences about the behavioural consequences of difficulty anticipation, although neural and psychophysiological evidence points to its motivational role in effort exertion. For example, in an fMRI study by Krebs and colleagues (2012) the brain areas supporting control-based attention were most activated in response to cues for a rewarded trial of higher effort level, suggesting a shared motivational effect of preparatory processing of reward and difficulty information (Krebs et al., 2012, see also Boehler et al., 2011). A study with an arithmetic task and separate reward and difficulty cueing blocks similarly found a shared network comprising of the anterior cingulate cortex (ACC) and the striatum supporting both cognitive effort preparation and prospective reward anticipation (Vassena et al., 2014). These studies suggest that the aversiveness of high difficulty before effort exertion is translated into a motivational signal when effort exertion is imminent. In addition, effort anticipation, like reward anticipation, has been shown to be indexed by pupil size, as pupil dilates more in anticipation of higher effort demands (Boehler et al., 2011). Yet, it is unclear whether, and how, this motivational value of anticipated difficulty influences effortful behaviour.

To probe effort regulation dynamics, we studied the effect of combined reward and difficulty cues with a short and long prediction horizon. By using two tasks, namely a random-dot-motion task (RDM, Experiments 1 and 2) and a Stroop task (Experiments 3 and 4), the generalisability of the effects across task-domains could also be gauged. In Experiments 1 and 3, each cue informed participants on the reward and difficulty level of the upcoming trial only (short prediction horizon), and this reward and difficulty level would hence randomly vary from one trial to the next. In Experiments 2 and 4, the information about reward and difficulty level was again signalled by a cue, but this information was valid for the upcoming six targets (long prediction horizon). Due to the facilitatory effect of reward in the Stroop task being widely reported in past work (e.g., Padmala and Pessoa, 2011; van den Berg et al., 2014), we expected to find a performance benefit for high compared to low reward conditions, which would be further amplified by a long prediction horizon. Compared to Stroop, RDM performance is more dependent on noisy sensory evidence accumulation rather than the allocation of cognitive control, which may result in more subtle effects of the reward manipulation. However, the RDM lends itself to a parametric manipulation of difficulty levels, determined by the percentage of coherently moving dots within the RDM stimulus. This feature of the RDM enabled interleaving intermediate difficulty trials with validly cued low and high difficulty trials to investigate the motivational influence of difficulty expectation on effort regulation independently of the actual difficulty level. The intermediate difficulty level was excluded in Experiments 3 and 4 as Stroop difficulty is categorical in nature (congruent versus incongruent) rather than continuous, so that an intermediate difficulty level would be easily detected by the participants thereby compromising the manipulation.

First, we expected to replicate the motivating effect of reward on effort allocation in both tasks, in that high reward should improve performance. This motivating effect of extrinsic reward might be further dependent on the difficulty level of the respective trial, as it has previously been shown that performance benefits were greater in a low difficulty condition (Krebs et al., 2012; Otto et al., 2022). Second, and most importantly, we predicted that the cost of effort regulation would be amortised by cues with a long prediction horizon (Experiments 2 and 4), resulting in larger cue-related adjustments in effort expenditure overall. Third, we expected to see similar modulations of effort allocation based on reward and prediction horizon in a subset of trials with fixed difficulty (i.e., intermediate difficulty trials), thereby probing *expected task difficulty* without the confounding factor of actual task difficulty. This prediction pertained only to Experiments 1 and 2, which included intermediate difficulty. Finally, we explored the potential contribution of inter-individual differences related to effort expenditure and reward-based motivation in the above processes. Specifically, it has been shown previously that the Need for Cognition trait (NFC) predicts incentivised effort expenditure (Sandra & Otto, 2018), and that reward responsiveness as measured by the BAS questionnaire (BIS-BAS; Carver & White, 1994) predicts more successful reward-related control adaptation (Braem et al., 2012). The data collection and analysis plan was preregistered for Experiment 1 (https://osf.io/qgehn), Experiment 2 (https://osf.io/9gkwu), and Experiment 3 and Experiment 4 (https://osf.io/27dwt).

## Experiments 1 & 2: Random-dot-motion task (RDM)

### Methods

#### Participants

49 participants were recruited for each Experiment, through the online research platform Prolific (https://www.prolific.co/). Participants performing below chance level (overall accuracy <55%) or failing to respond to more than 25% of the trials were excluded from the analyses, resulting in the final sample of 45 participants for Experiment 1 (22 female, 26 male; median age 21 years), and 49 participants for Experiment 2 (23 female, 26 male; median age 23). All participants had normal or corrected-to-normal vision and gave informed consent. Participants received £6,25 (GBP) as a base payment for taking part in the experiment, as well as a monetary bonus based on their performance on the task. The research protocol was approved by the Ethical Committee of the Faculty of Psychology and Educational Science at Ghent University.

#### Stimuli and procedure

**Figure 1:**
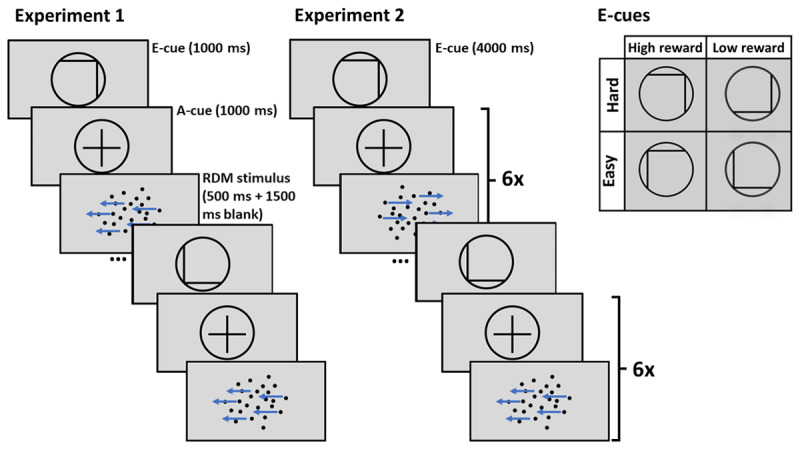
Trial structure of Experiment 1 with short prediction horizon, and Experiment 2 with long prediction horizon. *Note*. E-cue stands for evaluation cue, a-cue stands for allocation cue. In Experiment 1 only one allocation cue and one RDM stimulus follow each evaluation cue. In Experiment 2 the allocation cue and RDM stimulus are repeated six times after each evaluation cue.

The design was very similar across both experiments (Figure 1), and both were programmed with JavaScript jsPsych library (version 6.1.0; de Leeuw, 2015). Everything within the experiments was presented on a grey background. Participants completed an RDM task created with the jsPsych ‘RDK’ plugin (Rajananda et al., 2018): a cloud of black dots was presented, with a portion of the dots moving in a coherent direction (left or right).^1^ The difficulty of the tasks was a function of the percentage of coherently moving dots, with easy trials consisting of dot motion stimuli with 50% of the dots moving in coherent direction, intermediate trials having 30% coherence of motion, and difficult trials having 20% coherence.^2^ Each stimulus was presented for 500 ms, followed by 1500 ms of blank screen. Participants’ responses were recorded as long as they occurred within the 2000 ms after target onset. Participants were encouraged to respond as fast as possible by indicating the perceived (or guessed) direction of the coherently moving dots by pressing either ‘C’ (left) or ‘N’ (right) key on their keyboard.

At the beginning of each trial (Experiment 1), or a miniblock of six trials (Experiment 2), an evaluation cue (e-cue) was presented, followed by an allocation cue (a-cue). In Experiment 2, an a-cue was repeated before each of the six targets within a miniblock. Both cues were presented for 1000 ms each in Experiment 1. In Experiment 2, e-cues were presented for 4000 ms to compensate for the fact that they were presented less often. The e-cue consisted of a grey circle with a black border, modelled after cues used by Vassena et al. (2019). Within the circle there were a vertical and a horizontal line, indicating reward (high or low) and difficulty (easy or hard), respectively. The meanings of vertical or horizontal lines were counterbalanced across the participants: the vertical line indicated either difficulty or reward (line on left side of circle indicating easy/low reward, and line on the right side indicating hard/high reward) and the horizontal line indicated the other cueing condition (line at the bottom indicating low reward/easy, line at the top indicating high reward/hard). The cue mappings remained unchanged for each participant across the Experiment. The allocation cue (a-cue) was a rearrangement of the building elements of the e-cue, with the horizontal and vertical lines brought together to form a black cross inside the circle. After the a-cue, a dot-motion stimulus appeared. E-cues that indicated easy trials were followed by an easy stimulus on two-thirds of the trials. Similarly, e-cues signalling difficult trials were followed by difficult trials on two-third of the trials. On the remaining one-third of the trials, both cue types were followed by intermediate difficulty trials, to study the effect of difficulty expectancy independent of actual difficulty. Half of the trials of each difficulty condition were high reward trials, while the other half were low reward trials. 5 pence was rewarded for a correct response in high reward trials, and 1 pence in low reward trials.

Although trials were presented in a random order, we implemented the constraints that the same number of easy, intermediate, and difficult trials were presented per every 24 trials. The design was fully orthogonal, as both reward conditions co-occurred with each difficulty level the same number of times. Similarly, the direction of the coherently moving dots (i.e., left or right) was balanced within a block of 24 trials. In Experiment 2, each miniblock of six trials contained two intermediate difficulty trials, and the order of intermediate difficulty trials within the miniblocks was further predetermined to ensure that an intermediate trial would appear in each position within the miniblock approximately same number of times.

After each block of 24 trials (i.e., or four miniblocks of six trials in Experiment 2) participants received feedback on their accuracy and reward they had accumulated in the last block. In order to reduce individual differences in perceived competency and accumulated reward processing, the feedback was slightly adjusted by centring it around 80%.^3^ This way, participants never received feedback of 100% accuracy. Specifically, if accuracy between 60–80% was achieved during a block, the feedback accuracy was increased toward 80%. If the actual accuracy was above 80%, the feedback accuracy was slightly less. Participants did not receive trial-wise performance feedback during the task.

Before the main experimental procedure, participants were familiarised with the meanings of the four e-cues. In order to achieve this, participants were informed about the meaning of the e-cues explicitly during the instructions, and were then presented with each of the four e-cues four times, totalling 16 e-cue presentations of 2000 ms. Each e-cue was followed by a question of either the reward or difficulty level of the cue. If participants responded incorrectly, the same e-cue was presented again for 2000 ms, followed by the same question. Only once a correct response was given, could the participant proceed to the next e-cue, otherwise the question was repeated. Participants had to answer correctly on first attempt to 80% of the cue questions in order to proceed past the e-cue practice. After completing the e-cue practice, the participants completed a practice block of the experiment, practicing the RDM task. Participants could only proceed to the experiment phase if they achieved 75% accuracy within a block. The practice block could be repeated up to three times. Participants could not proceed to the experiment if 75% accuracy was not reached within these three attempts.

The main experimental procedure consisted of 12 blocks of 24 trials, each comprising of an e-cue, a-cue, and a dot-motion stimulus. The different e-cues were presented in random order, but balanced within a block of 24 trials. This implied that in Experiment 2, each of the four miniblock conditions was presented once per 24 trials (i.e., four miniblocks of six trials).

At the end of the experiment, participants received feedback of their overall accuracy and reward, after which they completed Likert scale style questionnaires about the likeability of each e-cue, and the demand associated with them. Finally, four questionnaires were administered after the experiment. These questionnaires measured trait differences that possibly add inter-individual variance in experimental manipulation effects. These questionnaires were the Need for Cognition (NFC, short version; Cacioppo et al., 1984), the Behavioral Inhibition System and Behavioral Activation System Questionnaire (BIS-BAS; Carver & White, 1994), Adult ADHD Self-Report Screening Scale for DSM-5 (ASRS-5; Ustun et al., 2017), and the Abbreviated Math Anxiety Scale (AMAS; Hopko et al., 2003). The AMAS was administered for comparability with another version of the experiment that used an arithmetic task instead of the RDM, and will not be further discussed in this paper. Similarly, the ASRS-5 was included for a planned analysis across different studies within a larger project and is not considered here.

#### Statistical analyses

Both experiments featured two within-participants factors, i.e., *reward* with two levels (high vs. low) and *difficulty* with three levels (easy vs. intermediate vs. hard). *Prediction horizon* (short Experiment 1 vs. long Experiment 2) was included as a between-participants factor with two levels. Both correctly cued and intermediate trials were thus modelled with a 2x2x2 model of each dependent measure, i.e., reaction times (RT) and accuracy. In analyses of correctly cued trials, actual *difficulty* (easy vs. hard) without invalidly cued intermediate trials was included as a factor. To test the effect of difficulty expectation, the analysis was restricted to intermediate difficulty trials, and the fixed effect of *difficulty cue* (easy-cued vs. hard-cued) replaced the fixed effect of *difficulty*. Both RT and accuracy were modelled with Generalized Linear Mixed Models (GLMMs) in which the main effects, and the interactions, of *difficulty, reward*, and *prediction horizon* were included as fixed effects. All factors across the main models had two levels each. These factors were sum contrast coded, where each factor level is compared to the grand mean of that factor. The maximal random effects structure that converged, determined with a step-wise procedure of the package ‘buildmer’ (Voeten, 2020), was used for each model, as per recommendation of Barr et al. (2013). In case of no convergence via the automated procedure, manually ran maximal random effects structure that converged was used. The fully maximal random effects structure tested included the main effects, and the interaction *reward* and *difficulty* within participants (i.e. allowing for random variability in the interaction of *reward* and *difficulty [cue]*, and their main effects, across participants). To test the effect of difficulty expectation, the analysis was restricted to intermediate difficulty trials, and the fixed effect of *difficulty cue* (easy-cued vs. hard-cued) replaced the fixed effect of *difficulty* (easy vs. hard). The structure of each model is reported in Tables 1, 2, 3, 4.

**Table 1:** *Output of a GLMM of RT across all difficulty levels with random effect structure* reward * difficulty | participant.


*PREDICTORS*	RT		

	*ESTIMATES*	*CI*	*p*

(Intercept)	542.84	510.30–577.46	**<0.001**

Reward (high)	1.00	0.99–1.00	0.213

Difficulty (easy)	0.95	0.94–0.96	**<0.001**

Experiment 2	1.01	0.95–1.08	0.671

Reward (high) * Difficulty (easy)	1.00	1.00–1.01	0.208

Reward (high) * Experiment 2	1.00	0.99–1.00	0.450

Difficulty (easy) * Experiment 2	1.00	0.99–1.01	0.529

Reward (high) * Difficulty (easy) * Experiment 2	1.00	0.99–1.01	0.874

**Random Effects**			

σ^2^	0.05		

τ_00 participant_	0.01		

τ_11 participant.Reward (__high)_	0.00		

τ_11 participant.Difficulty (easy)_	0.00		

τ_11 participant.__Reward (high):Difficulty (easy)_	0.00		

ρ_01_	–0.09		

	–0.06		

	0.02		

ICC	0.17		

N _participant_	94		

Observations	14780		

Marginal R^2^/Conditional R^2^	0.047/0.205		


**Table 2:** *Output of GLMM of accuracy across all difficulty levels with random effect structure* difficulty | participant.


*PREDICTORS*	ACCURACY		

	*ODDS RATIOS*	*CI*	*p*

(Intercept)	6.78	5.57–8.26	**<0.001**

Reward (high)	1.03	0.99–1.08	0.162

Difficulty (easy)	2.11	1.92–2.33	**<0.001**

Experiment 2	0.95	0.78–1.15	0.577

Reward (high) * Difficulty (easy)	1.03	0.99–1.08	0.147

Reward (high) * Experiment 2	1.05	1.00–1.09	**0.038**

Difficulty (easy) * Experiment 2	1.02	0.93–1.12	0.699

Reward (high) * Difficulty (easy) * Experiment 2	1.05	1.01–1.10	**0.028**

**Random Effects**			

σ^2^	3.29		

τ_00 participant_	0.86		

τ_11 participant.Difficulty (easy)_	0.14		

ρ_01_	0.90		

ICC	0.23		

N _participant_	94		

Observations	18050		

Marginal R^2^/Conditional R^2^	0.117/0.322		


**Table 3:** *Output of a GLMM of RT in intermediate difficulty trials with random effect structure* reward * difficulty cue | participant.


*PREDICTORS*	RT		

	*ESTIMATES*	*CI*	*p*

(Intercept)	547.44	513.11–584.08	**<0.001**

Reward (high)	1.00	0.99–1.01	0.899

Difficulty cue (easy)	0.99	0.99–1.00	0.117

Experiment 2	1.02	0.96–1.09	0.563

Reward (high) * Difficulty cue (easy)	1.00	0.99–1.01	0.726

Reward (high) * Experiment 2	1.00	0.99–1.01	0.893

Difficulty cue (easy) * Experiment 2	0.99	0.99–1.00	0.184

Reward (high) * Difficulty cue (easy) * Experiment 2	1.00	0.99–1.01	0.676

**Random Effects**			

σ^2^	0.05		

τ_00 participant_	0.01		

τ_11 participant.Reward (high)_	0.00		

τ_11 participant.Difficulty cue (easy__)_	0.00		

τ_11 participant. Reward (high): Difficulty cue (easy)_	0.00		

ρ_01_	–0.05		

	0.01		

	0.11		

ICC	0.52		

N _participant_	94		

Observations	7413		

Marginal R^2^/Conditional R^2^	0.007/0.204		


**Table 4:** *Output of a GLMM of accuracy in intermediate difficulty trials with random effect structure* reward | participant.


*PREDICTORS*	ACCURACY		

	*ODDS RATIOS*	*CI*	*p*

(Intercept)	5.83	4.83–7.03	**<0.001**

Reward (high)	1.01	0.93–1.09	0.883

Difficulty cue (easy)	1.03	0.97–1.09	0.341

Experiment 2	0.95	0.79–1.15	0.618

Reward (high) * Difficulty cue (easy)	1.07	1.01–1.13	**0.019**

Reward (high) * Experiment 2	0.95	0.89–1.02	0.170

Difficulty cue (easy) * Experiment 2	1.06	1.00–1.12	**0.046**

Reward (high) * Difficulty cue (easy) * Experiment 2	1.06	1.00–1.12	0.061

**Random Effects**			

σ^2^	3.29		

τ_00 participant_	0.75		

τ_11 participant. Reward (high)_	0.03		

ρ_01_	0.15		

ICC	0.19		

N _participant_	94		

Observations	9020		

Marginal R^2^/Conditional R^2^	0.004/0.195		


All the models were fitted in R 4.0.4 (R Core Team, 2021) in conjunction with RStudio 2022.07.1, using the ‘lme4’ package (Bates et al., 2015). Wald test statistics were obtained using the ‘car’ package (Fox & Weisberg, 2019), with statistical significance level set to *p* < .05. In case of significant interaction effects the ‘emmeans’ package (Lenth, 2018) was used for pairwise comparisons. Follow-up tests of main and interaction effects are corrected for multiple comparisons with False Discovery Rate (FDR).

Accuracy was fitted with a binomial distribution and a logit link function (logistic regression) across all experiments. RT of correct responses (slower than 200 ms and faster than the participant- and cue-wise mean RT plus 2.5 standard deviations) were fitted with a Gamma distribution and a log link function (log-transformation of the means) across RT analyses.

According to Brysbaert and Stevens’ recommendation, 1600 observations are required for a mixed effects model to have sufficient power to detect small effects that are typical of RT effects (Cohen’s d 0.2; Brysbaert & Stevens, 2018). After exclusions, each of the (validly cued) conditions that are included in the RT analysis comprise of 1677 to 1998 trials. The accuracy analysis has even more power, as accuracy effects are generally larger and the analysis includes both incorrect and correct trials. Analyses of reward and difficulty cueing effects in intermediate difficulty trials are underpowered with 880 to 956 correct trials per condition. These results should thus be regarded with caution.

To further explore whether reward cueing effects change during the course of miniblocks in Experiment 2, we also analysed the effect of trial number within a miniblock with all difficulty levels included. Trial number was added as a factor with six levels that were Helmert contrast coded. Helmert contrast coding is used to test whether an effect is significantly different between one level and the mean of the subsequent levels (Schad et al., 2020). This way, we could test whether cues were effective across the miniblock, or whether they were building up over time. The random effect structure remained the same as in the main analysis.

Finally, to explore the influence of inter-individual differences, the questionnaire scores (i.e., the NFC and the reward responsiveness subscale of the BIS-BAS) were z-transformed and added as a continuous predictor in separate GLMMs to those described above, keeping the rest of the model structure identical where possible. Additionally, likeability and demand ratings of each of the cues were compared separately using a two-way ANOVA (*reward* × *difficulty*).

### Results

#### The effect of task difficulty on performance (manipulation check)

Across both studies increasing difficulty (decreasing coherence) was associated with decreased accuracy and increased RT, attesting a successful difficulty manipulation. Specifically, participants were faster to respond to easy targets, compared to intermediate difficulty targets (Experiment 1: *b* = –0.05, SE = 0.01, *z* = –4.88, *p* < .0001; Experiment 2: *b* = –0.07, SE = 0.01, *z* = –6.99, *p* < .0001), as well as to the hard targets (Experiment 1: *b* = –0.09, SE = 0.02, *z* = –5.71, *p* < .0001; Experiment 2: *b* = –0.11, SE = –0.02, *z* = –6.95, *p* < .0001). Additionally, responses to intermediate difficulty targets were faster than those to hard targets (Experiment 1: *b* = –0.04, SE = 0.01, *z* = –4.02, *p* < .0001; Experiment 2: *b* = –0.04, SE = 0.01, *z* = –3.72, *p* < .001). Similarly, accuracy was higher for easy targets compared to intermediate difficulty targets (Experiment 1: *b* = 0.56, SE = 0.06, *z* = 8.75, *p* < .0001; Experiment 2: *b* = 0.70, SE = 0.06, *z* = 10.88, *p* < .0001) and hard targets (Experiment 1: *b* = 1.03, SE = 0.06, *z* = 16.78, *p* < .0001; Experiment 2: *b* = 1.22, SE = 0.06, *z* = 19.90, *p* < .0001). Further, accuracy for intermediate difficulty targets was higher compared to hard targets (Experiment 1: *b* = 0.47, SE = 0.06, *z* = 8.54, *p* < .0001; Experiment 2: *b* = 0.53, SE = 0.05, *z* = 9.87, *p* < .0001).

#### The effect of prediction horizon and reward cues on effort allocation (including all difficulty levels)

**Figure 2:**
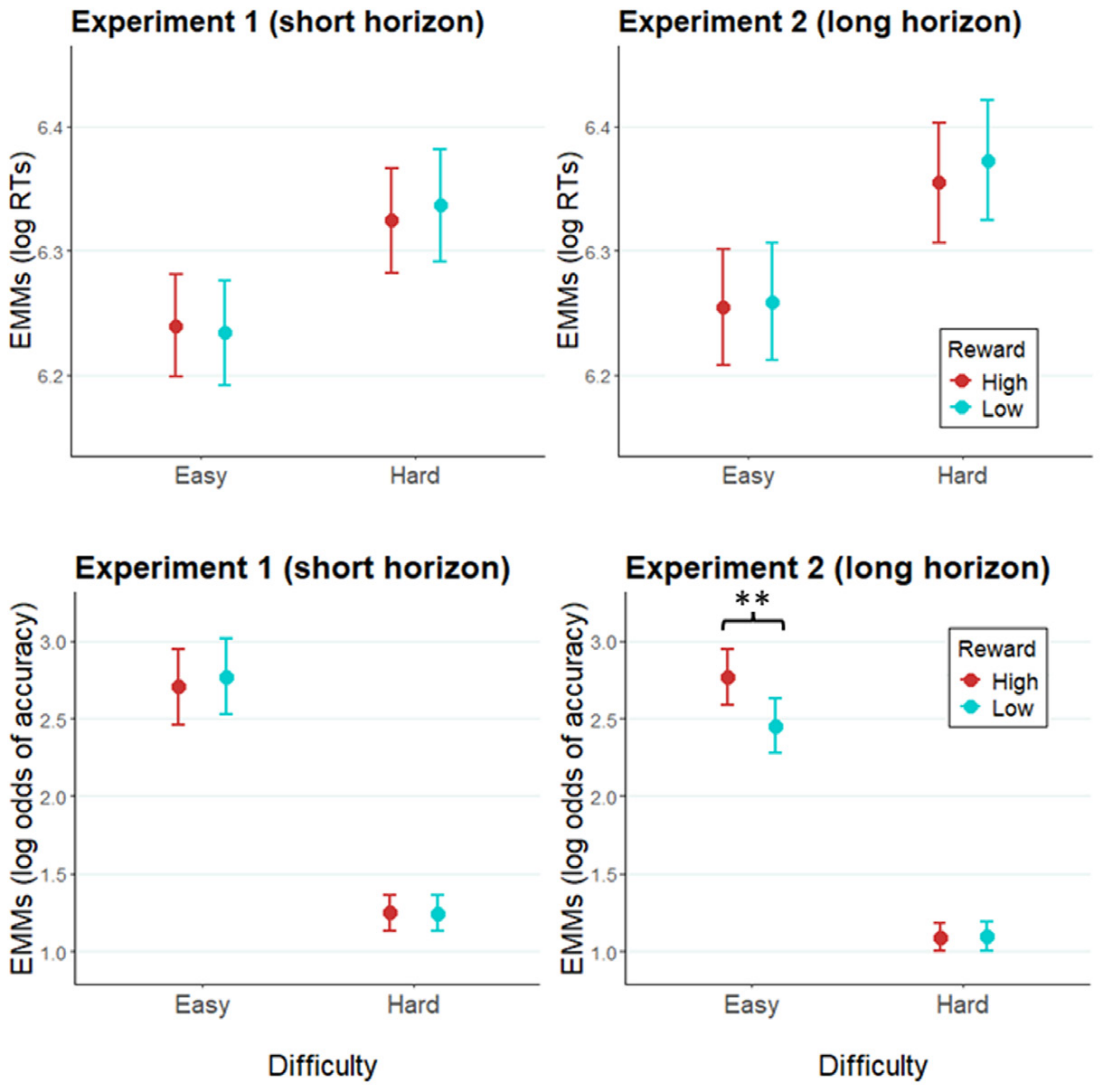
Modelled performance indices across easy and hard trials. *Note*. Estimated marginal means of log RT and of log odds of accuracy in Experiment 1 (left panel), and Experiment 2 (right panel). Error bars represent the standard error of the estimated marginal mean. ** = *p* < .01.

In accordance with neuroeconomic frameworks of effort allocation, we expected to find a facilitative effect of high versus low reward expectancy on RT and/or accuracy. A GLMM of RT revealed no significant effects of *reward* (*X^2^*[1] = 1.55, *p* = .21), *prediction horizon* (*X^2^*[1] = 0.18, *p* = .67), *reward × difficulty* (*X^2^*[2] = 1.58, *p* = .21), or *reward × difficulty × prediction horizon* (*X^2^*[2] = 0.03, *p* = .87; Figure 2). See Table 1 for random effects structure of the model, and full report of the fixed effects. A GLMM of accuracy (Table 2) revealed a significant *reward × prediction horizon* interaction (*X^2^*[1] = 4.33, *p* = .04), and a *reward × difficulty × prediction horizon* interaction (*X^2^*[1] = 4.86, *p* = .03). Specifically, Experiment 2 (using cues with a longer prediction horizon, Table S1 in Supplementary materials) featured a significant main effect of reward (*X^2^*[1] = 6.02, *p* = .01) and an interaction *reward* × *difficulty* (*X^2^*[2] = 6.64, *p* = .01), both of which were not observed in Experiment 1 (*reward*: *X^2^*[1] = 0.29, *p* = .63; *reward* × *difficulty*: *X^2^*[1] = 0.29, *p* = .59; Table S2 in Supplementary materials). Follow-up tests in Experiment 2 showed that accuracy was higher following high versus low reward cues across both difficulties (*b* = 0.15, SE = 0.06, *z* = 2.45, *p* = .01). Further, the simple effect of reward was significant in easy trials (*b* = 0.31, SE = 0.10, *z* = 3.02, *p* < .01), but not in hard trials (*b* = –0.01, SE = 0.07, *z* = –0.11, *p* = .91). The main effect of *prediction horizon* was not significant in accuracy (*X^2^*[1] = 0.31, *p* = .58).

#### Follow-up analysis: The effect of trial number on effort allocation in Experiment 2

**Figure 3:**
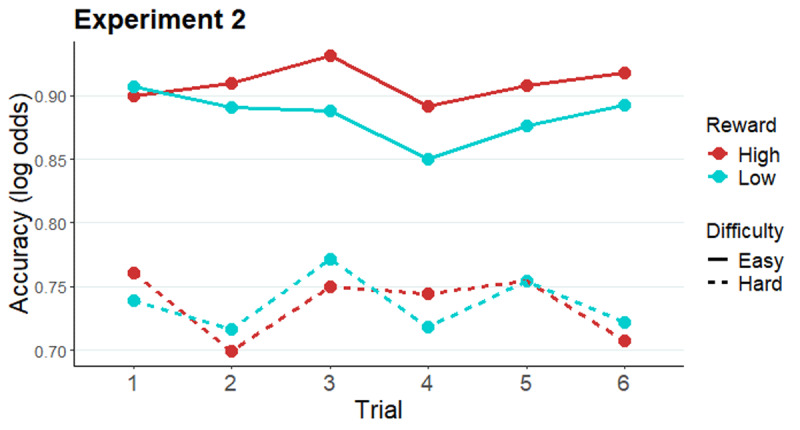
Modelled performance indices across all six trials after an evaluation-cue (easy and hard conditions). *Note*. Estimated marginal means of log odds of accuracy along the miniblock of six trials preceded by an evaluation-cue in Experiment 2.

To test whether the above-reported cueing effects on accuracy observed in Experiment 2 were effective immediately after cue onset or increased gradually over time, the accuracy data of Experiment 2 were fitted with a GLMM, with *reward* (high, low), *difficulty* (easy, intermediate, hard), and *trial* (factor, levels 1–6) as fixed effects (for a summary of each term of the model, see Table S3 in Supplementary materials). Note that this analysis focuses on the potential impact of the additional variable (*trial*) on the effects observed in the original analysis above. As expected, the two-way interaction between reward cue and difficulty was replicated when the predictor variable of trial number within miniblock is added in the model (*X^2^*[2] = 5.88, *p* = .02; Figure 3). Importantly, this effect was not modulated by trial number, as the three-way interaction *reward* × *difficulty* × *trial* was not significant (*X^2^*[10] = 4.88, *p* = .43). We note that the reward by difficulty interaction was not significant on any individual trial, but that this effect was significant when collapsing accuracy data across trials. The main effect of trial was marginally significant (*X^2^*[5] = 10.04, *p* = .07).

#### The effect of prediction horizon, reward, and difficulty expectation on effort allocation when task difficulty is fixed (intermediate difficulty level)

**Figure 4:**
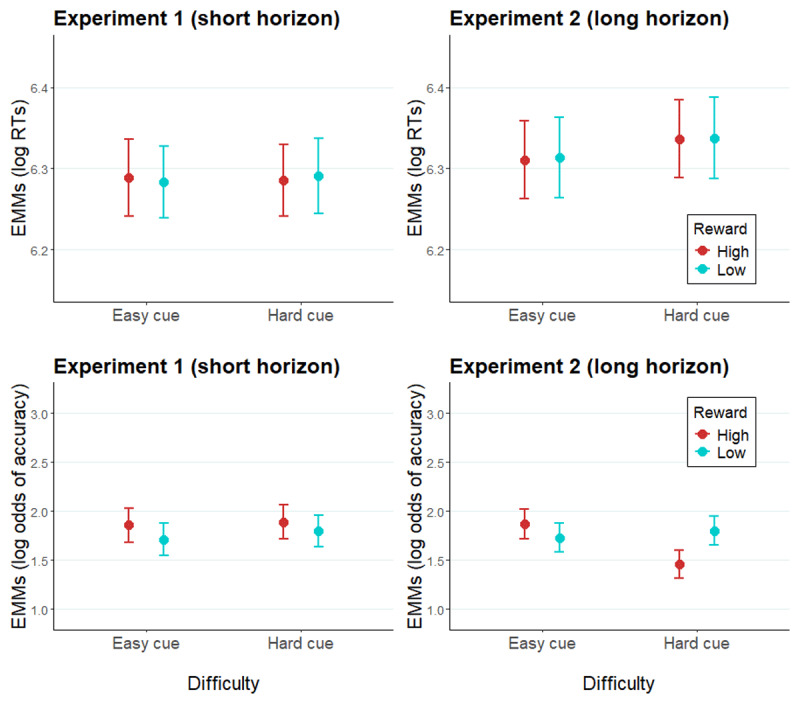
Modelled performance indices of intermediate difficulty trials in Experiment 1 and Experiment 2. *Note*. Estimated marginal means of log RTs and log odds of accuracy of intermediate difficulty trials in Experiment 1 (left panel) and Experiment 2 (right panel). Error bars represent the standard error of the estimated marginal means.

Zooming in on intermediate difficulty trials (i.e., targets of identical difficulty preceded by hard versus easy cues), we tested whether participants also modulate effort allocation in accordance with *expected* difficulty without the confounding effect of actual target difficulty. This also provides another perspective on the impact of the prediction horizon, but now based on expected rather than actual difficulty. To this end, RT data of intermediate difficulty trials were fitted with GLMMs with fixed effects of *reward, difficulty cue* (note, not actual difficulty, which is now constant), and *prediction horizon* (Figure 4; Table 3). No significant main effects or interactions were found in RTs.

An analogous GLMM of the accuracy data (Table 4) showed a significant two-way *difficulty cue* × *prediction horizon* interaction (*X^2^*[1] = 3.98, *p* = .05), indicating that trials cued as easy were responded to more accurately than those cued hard, but only in Experiment 2 (*b =* 0.17, SE = 0.08, *z* = 2.11, *p* = .04; Experiment 1: *b =* –0.06, SE = 0.08, *z* = –0.73, *p* = .47). A *reward* × *difficulty cue* interaction was additionally significant (*X^2^*[1] = 5.54, *p* = .02). Follow-up tests revealed non-significant reverse effects of reward within the two difficulty conditions across the experiments. Paralleling our main findings, numerically, accuracy is facilitated by higher reward in easy cue condition (*b =* 0.15, SE = 0.10, *z* = 1.51, *p* = .13), whereas in hard cue condition high reward has a deleterious effect on accuracy (*b = –*0.12, SE = 0.10, *z* = –1.28, *p* = .20). A marginally significant three-way *reward* × *difficulty cue* × *prediction horizon* interaction (*X^2^*[1] = 3.52, *p* = .06) indicates that this effect might be driven by Experiment 2: pairwise comparisons showed a significant, counterintuitive effect of *reward* when a hard trial was expected in Experiment 2, as high reward cues were associated with lower accuracy compared to low reward cues (*b =* –0.33, SE = 0.13, *z* = 2.58, *p* = .01). Again, the main effect of *prediction horizon* was not significant (*X^2^*[1] = 0.25, *p* = .62). We note that the interaction between reward and expected difficulty, numerically, was in the same direction as the main analysis on actual (rather than expected) difficulty. Although the exact pattern seems slightly different, we should remain cautious about interpreting this pattern as this analysis was underpowered due to being performed on only a small subset of the data.

#### Inter-individual differences in reward responsiveness and need for cognition (NFC)

In addition to these global effort regulation dynamics, we sought to investigate the possible influence of inter-individual differences. To this end, RT and accuracy data were modelled with GLMMs with fixed effects of the within-participant factors *reward* and *difficulty*, and *prediction horizon* as a between-participants factor, and z-transformed questionnaire scores added as a continuous predictor variable. In follow-up tests the questionnaire scores were split into high (1 standard deviation above the mean), mean, and low (1 standard deviation below the mean) scores.

No significant main effect of, or interaction effects with, *reward responsiveness* were uncovered in RTs or accuracy. The same models were tested with *NFC* scores added as a continuous predictor (Table S6 in Supplementary materials). A significant three-way interaction *reward* × *difficulty* × *NFC* in RTs (*X^2^*[1] = 11.06, *p* < .001), showing a reward benefit in hard trials in people high in NFC (*b =* –0.03, SE = 0.01, *z* = –3.70, *p* < .001). No significant effects of *NFC* were uncovered in accuracy (Table S7 in Supplementary materials).

#### Likeability and demand rating of the cues

**Figure 5:**
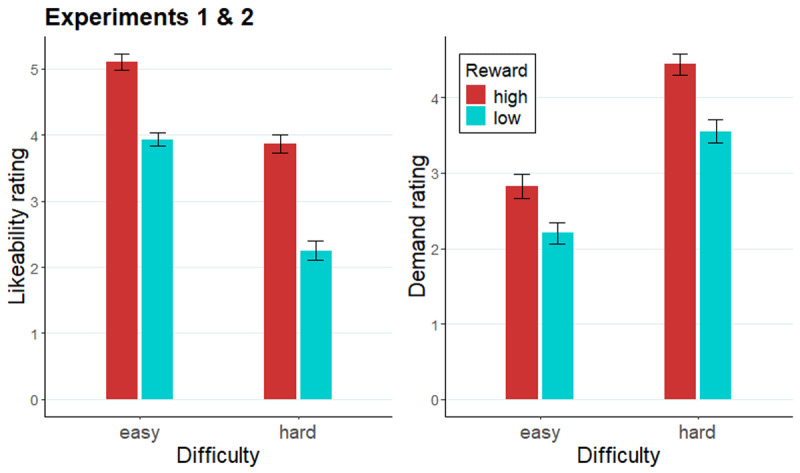
Likeability and demand ratings of evaluation cues across Experiments 1 and 2. *Note*. Likeability (left panel) and demand (right panel) rating of evaluation cues across Experiment 1 and 2. Error bars represent the standard error of the mean.

Last, we evaluated the likeability and demand ratings of the four e-cues across the two experiments (Figure 5). As expected, participants rated the high reward cues (*M* = 4.49) to be more likeable than low reward cues (*M* = 3.10; *F*[1, 93] = 96.50, *p* < .001; collapsed across difficulty), and easy cues (*M* = 4.53) as more likeable than hard cues (*M* = 3.06; *F*[1, 93] = 114.95, *p* = .001; collapsed across reward). Additionally, a *reward* × *difficulty* interaction was significant in the cue likeability ratings (*F*[1, 93] = 6.84, *p* = .01), indicating the difference in likeability ratings of high and low reward cues within hard cues was larger (*b =* 1.62, SE = 0.19, *t* = 8.73, *p* < .0001) than within easy cues (*b =* 1.17, SE = 0.14, *t* = 8.17, *p* < .0001). High reward cues (*M* = 3. 63) were rated as more demanding than low reward cues (*M* = 2.88), and hard cues (*M* = 4.00) as more demanding than easy cues (*M* = 2.51). Additionally, a *reward* × *difficulty* interaction was significant in the cue demand ratings (*F*[1, 93] = 9.25, *p* = .003), indicating the difference in demand rating for easy compared to hard cues was larger when these were coupled with low rather than high reward.

## Experiments 3 & 4: Stroop task

### Methods

#### Participants

50 participants were recruited through Prolific for each experiment. The inclusion and exclusion criteria were identical to Experiments 1 and 2, resulting in a final sample of 50 participants for Experiment 3 analyses (24 women, median age 24 years), and 48 participants for Experiment 4 analyses (23 women, median age 25 years). Participants received £6,75 (GBP) as a base payment for taking part in the experiment (increased from base payment of £6,25 in Experiments 1 and 2 in accordance with updated recommendation by Prolific by the time of Experiments 3 and 4), and an additional performance-dependent bonus.

#### Stimuli and procedure

**Figure 6:**
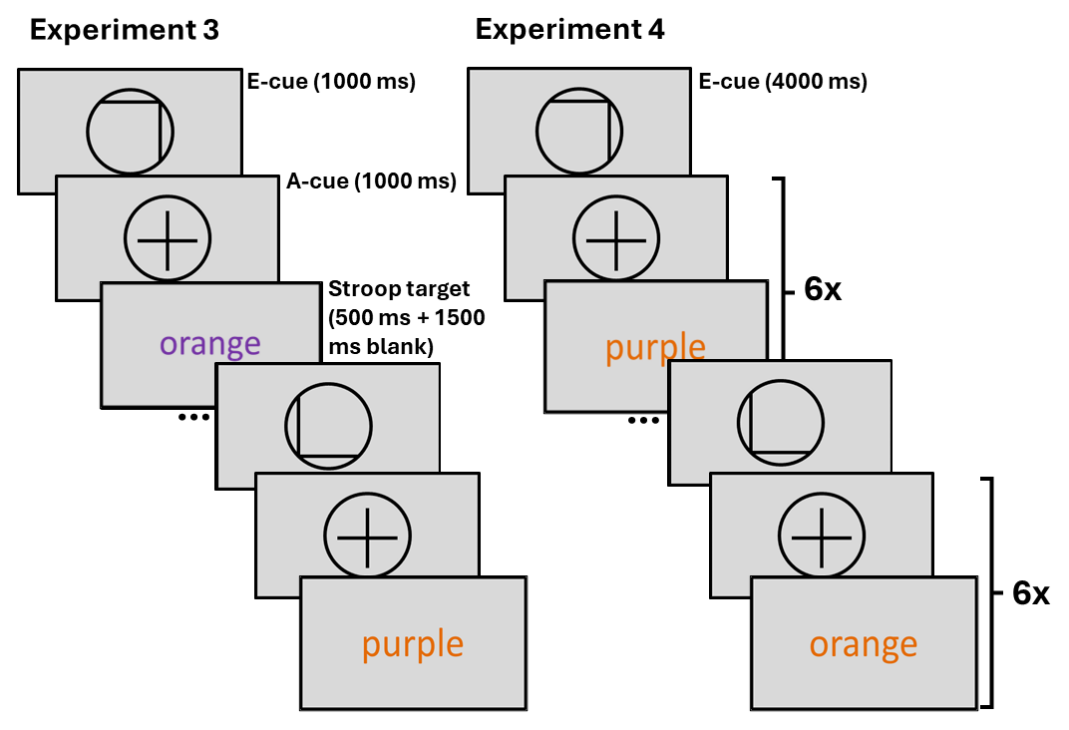
Trial structure of Experiment 3 with short prediction horizon, and Experiment 4 with long prediction horizon. *Note*. E-cue stands for evaluation cue, a-cue stands for allocation cue. In Experiment 3 only one allocation cue and one Stroop target follow each evaluation cue. In Experiment 4 the allocation cue and a varying Stroop target of the cued difficulty (congruency) are presented six times after each evaluation cue.

The design of Experiments 3 and 4 was identical to the design of Experiments 1 and 2, apart from the differences specified here (Figure 6). First notable difference was the change of task from the RDM to the Stroop task (Stroop, 1935). By pressing either key ‘C’ or key ‘N’ on their keyboard, participants indicated whether the Stroop target word was written in orange (RGB: 228, 108, 10) or purple (RGB: 112, 48, 160) ink. In easy trials the Stroop target was congruent, with the ink colour matching the meaning of the word. In hard trials the Stroop target was incongruent, as the ink colour did not match the meaning of the word. In each trial, the Stroop target word was presented for 500 ms, followed by 1500 ms of blank screen, during which responses were still recorded. Another notable deviation from the design of the RDM experiments was the exclusion of intermediate difficulty trials. Here, the e-cues were 100% informative, as each ‘easy’ e-cue was followed by (an) easy trial(s), and each ‘hard’ e-cue by (a) hard trial(s, see Introduction for rationale). Again, the reward and difficulty levels were counterbalanced across each block of 24 trials (Experiment 3) or across four miniblocks of 6 trials (Experiment 4). Previous work has reported generally high accuracy in the Stroop task (e.g. van den Berg et al., 2014), which was projected to be even higher in the current experiments due to limiting the response options to two (in contrast to previous manual Stroop tasks with three or four response options). Therefore, unlike in Experiments 1 and 2, the participants could proceed to the experiment regardless of their accuracy after completing one practice block of the task.

After completing the task, participants rated the likeability and demand of the e-cues, and completed the same questionnaires as in Experiments 1 and 2, with the exception of the Abbreviated Math Anxiety Scale (AMAS; Hopko et al., 2003), which was excluded. Again, ASRS-5 results are not discussed.

#### Statistical analyses

Both experiments featured two within-participants factors, i.e., *reward* with two levels (high vs. low) and *difficulty* with two levels (easy vs. hard; no intermediate difficulty). *Prediction horizon* (short in Experiment 3 vs. long in Experiment 4) was included as a between-participants factor with two levels, resulting in a 2x2x2 model for RT and accuracy.

As in Experiments 1 and 2, accuracy was fitted with a binomial distribution and a logit link function (logistic regression) across both experiments. RT of correct responses were fitted with a Gamma distribution. The models are reported in full in Tables 5 and 6.

**Table 5:** *Output of a GLMM of RT across all difficulty levels with random effect structure* 1 | participant.


*PREDICTORS*	RT		

	*ESTIMATES*	*CI*	*p*

(Intercept)	405.23	393.00–417.84	**<0.001**

Reward (high)	1.00	0.99–1.00	**<0.001**

Difficulty (easy)	0.99	0.99–0.99	**<0.001**

Experiment 4	0.99	0.96–1.02	0.592

Reward (high) * Difficulty (easy)	1.00	1.00–1.00	0.345

Reward (high) * Experiment 4	1.00	1.00–1.00	**0.039**

Difficulty (easy) * Experiment 4	1.00	1.00–1.00	0.249

Reward (high) * Difficulty (easy) * Experiment 4	1.00	1.00–1.00	0.510

**Random Effects**			

σ^2^	0.03		

τ_00 participant_	0.00		

ICC	0.06		

N _participant_	98		

Observations	25547		

Marginal R^2^/Conditional R^2^	0.005/0.068		


**Table 6:** *Output of a GLMM of accuracy across all difficulty levels with random effect structure* difficulty | participant.


*PREDICTORS*	ACCURACY		

	*ODDS RATIOS*	*CI*	*p*

(Intercept)	20.25	17.19–23.85	**<0.001**

Reward (high)	1.04	0.99–1.10	0.130

Difficulty (easy)	1.18	1.10–1.26	**<0.001**

Experiment 4	1.21	1.03–1.42	**0.023**

Reward (high) * Difficulty (easy)	1.03	0.98–1.09	0.226

Reward (high) * Experiment 4	1.02	0.96–1.07	0.538

Difficulty (easy) * Experiment 4	1.02	0.96–1.09	0.437

Reward (high) * Difficulty (easy) * Experiment 4	1.00	0.95–1.05	0.970

**Random Effects**			

σ^2^	3.29		

τ_00 participant_	0.58		

τ_11 participant__.Difficulty (easy)_	0.02		

ρ_01 participant_	–0.74		

ICC	0.16		

N _participant_	98		

Observations	27233		

Marginal R^2^/Conditional R^2^	0.016/0.169		


With each of the conditions across the two experiments including 3134 to 3266 correct trials, the design has high power for detection of small effect sizes (Brysbaert & Stevens, 2018).

### Results

#### The effect of task difficulty on performance (manipulation check)

Across the two Stroop experiments, easy trials were responded to faster than hard trials (*X^2^*[1] = 4.88, *p* < .0001; Experiment 3: *b* = –0.01, SE = 0.003, *z* = –4.14, *p* < .0001; Experiment 4: *b* = –0.02, SE = 0.003, *z* = –5.74, *p* < .0001). Easy trials also yielded higher accuracy than hard trials (*X^2^*[1] = 21.11, *p* < .0001; Experiment 3: *b* = 0.28, SE = 0.09, *z* = 3.04, *p* < .01; Experiment 4: *b* = 0.37, SE = 0.10, *z* = 3.77, *p* < .001).

#### The effect of prediction horizon and reward cues on effort allocation

**Figure 7:**
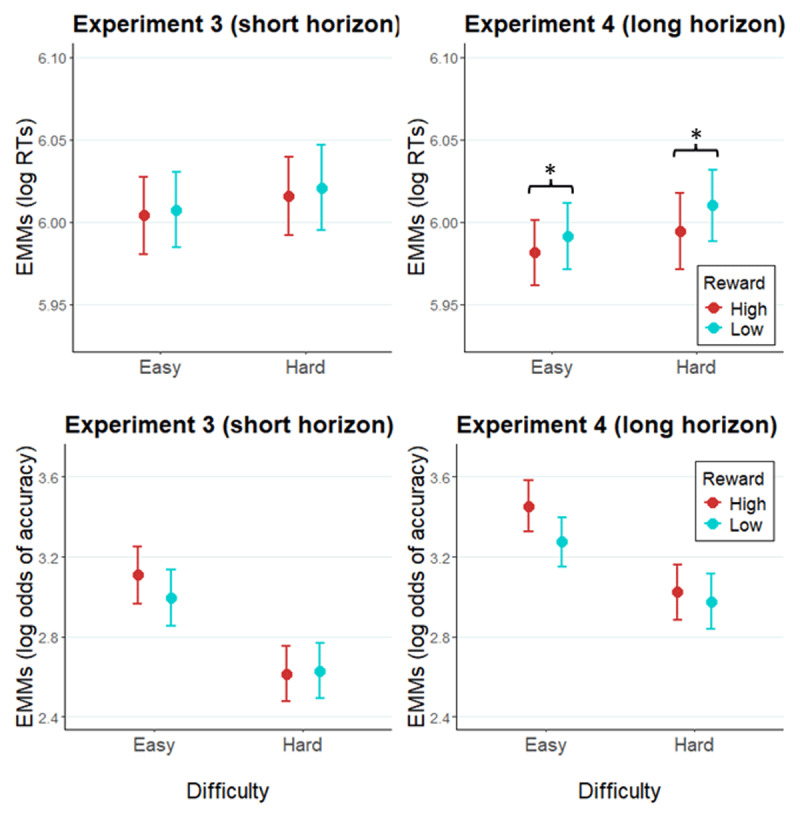
Modelled performance indices in Experiment 3 and Experiment 4. *Note*. Estimated marginal means of RTs and of log odds of accuracy in Experiment 3 (left panel), and Experiment 4 (right panel). Error bars represent the standard error of the estimated marginal mean. * = *p* < .05.

As in the RDM experiments, we expected to find a facilitative effect of high reward on performance. Additionally, we expected to replicate the RDM finding that longer prediction horizon leads to a stronger impact of reward expectancy on performance. A *reward × difficulty × prediction horizon* GLMM of RT (Figure 7; see Table 5 for a detailed summary of model terms) revealed a significant main effect of *reward* (*X^2^*[1] = 1.72, *p* < .0001), as high reward led to faster RTs (*b* = –0.009, SE = 0.002, *z* = –4.15, *p* < .0001). Importantly, the *reward* × *prediction horizon* interaction (*X^2^*[1] = 4.25, *p* = .04) was also significant. Separate analyses of the two experiments (Experiment 4: Table S8 in Supplementary materials, Experiment 3: Table S9 in Supplementary materials) showed a significant reward effect in Experiment 4 (*X^2^*[1] = 6.43, *p* = .01) where high reward was associated with faster RT than low reward (*b* = –0.01, SE = 0.01, *z* = –2.54), whereas no significant reward effect was found in Experiment 3 (*X^2^*[1] = 0.88, *p* = .35). A GLMM of accuracy (Table 6) revealed a significant main effect of *prediction horizon* (*X^2^*[1] = 5.14, *p* = .02), as accuracy was higher in Experiment 4 (*b* = 0.38, SE = 0.17, *z* = 2.27).

#### Follow-up analysis: The effect of miniblock target number on effort allocation in Experiment 4

**Figure 8:**
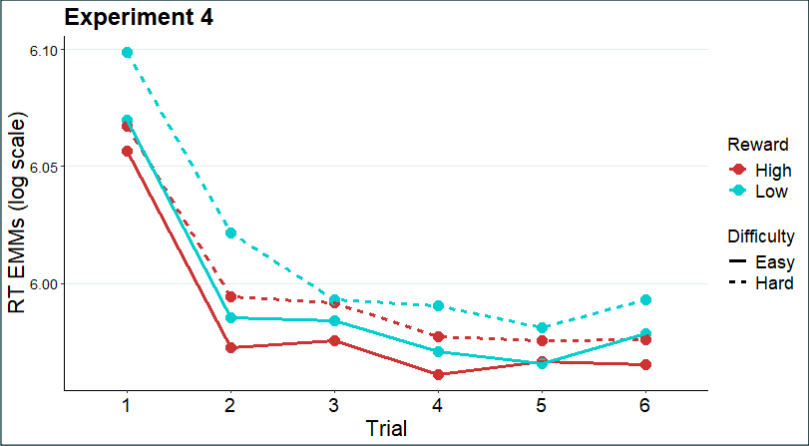
Modelled reaction times across trials of a miniblock in Experiment 4. *Note*. Estimated marginal means of RT along the miniblock of six trials preceded by an evaluation-cue in Experiment 4.

As in Experiment 2, we tested whether the reward effect in Experiment 4 with long prediction horizon evolved with target number within miniblock, or if this effect exists from the beginning of the miniblock. Adding the factor of *trial* (1–6) within miniblock into a GLMM of Experiment 4 RT (Table S10 in Supplementary materials), the main effect of *reward* remained significant (*X^2^*[1] = 19.35, *p* < .0001; Figure 8). Importantly, the effect of reward was not modulated by trial number, as the interaction *reward* × *trial* was not significant (*X^2^*[5] = 6.36, *p* = .27). For completeness, there was a significant main effect of trial (*X^2^*[5] = 562.89, *p* < .0001). Each trial was responded to faster than the mean of the previous trials (all *b*s = –0.03– –0.08, all SEs = 0.004 – 0.005, *z*s = –5.68– –15.75, all *p*s < .0001).

#### Inter-individual differences in reward responsiveness and need for cognition (NFC)

Again, in addition to generalisable effects of reward, difficulty, and prediction horizon, exploratory analyses were carried out to investigate individual differences in these effects. The questionnaire scores of reward responsiveness and NFC were added in separate GLMMs as continuous predictor variables.

A significant *reward* × *prediction horizon* × *reward responsiveness* interaction was found in RT (*X^2^*[1] = 4.07, *p* = .04; Table S11 in Supplementary materials). In Experiment 4, participants low in reward responsiveness did not exhibit a facilitatory effect of high reward in RTs in easy trials (*b =* –0.0002, SE = 0.005, *z* = –0.03, *p* = .97), but did in hard trials (*b =* –0.01, SE = 0.006, *z* = –2.27, *p* = .02). Participants with intermediate and high levels of reward responsiveness had a reward effect in RTs across difficulty levels. Apart from a marginal reward effect in low reward responsiveness in easy trials (*b =* –0.01, SE = 0.006, *z* = –1.69, *p* = .09), no significant reward effects were found at any level of reward responsiveness in Experiment 3 (all *p*s > .11). In accuracy (Table S12 in Supplementary materials), a *reward* × *difficulty* × *reward responsiveness* interaction was significant (*X^2^*[1] = 4.65, *p* = .03). Across both Stroop experiments, high (*b =* 0.27, SE = 0.12, *z* = 2.22, *p* = .03) and intermediate (*b =* 0.16, SE = 0.08, *z* = 1.93, *p* = .05) *reward responsiveness* were associated with increased accuracy in highly rewarded easy trials compared to low reward easy trials, parallelling the main finding in Experiment 2.

Adding *NFC* into the RT model as a predictor variable (Table S13 in Supplementary materials), a significant *reward* × *prediction horizon* × *NFC* interaction was uncovered (*X^2^*[1] = 4.51, *p* = .03): no reward effect was found across NFC levels in Experiment 3, while in Experiment 4 only those with high and intermediate levels of NFC had a significant boosting effect of reward on their RT (high NFC: *b =* –0.02, SE = 0.004, *z* = –4.90, *p* < .0001; intermediate NFC: *b =* –0.01, SE = 0.003, *z* = –4.50, *p* < .0001). In accuracy, *NFC* interacted with *difficulty* and *prediction horizon* (*X^2^*[1] = 4.17, *p* = .04). Difficulty had an impact on accuracy across NFC levels (all *p*s < .01), apart from in low NFC in Experiment 3 (*b =* 0.12, SE = 0.12, *z* = 0.97, *p* = .33), and the effect of difficulty is only marginally significant in high NFC in Experiment 4 (*b =* 0.27, SE = 0.14, *z* = 1.89, *p* = .06).

#### Likeability and demand rating of the cues

**Figure 9:**
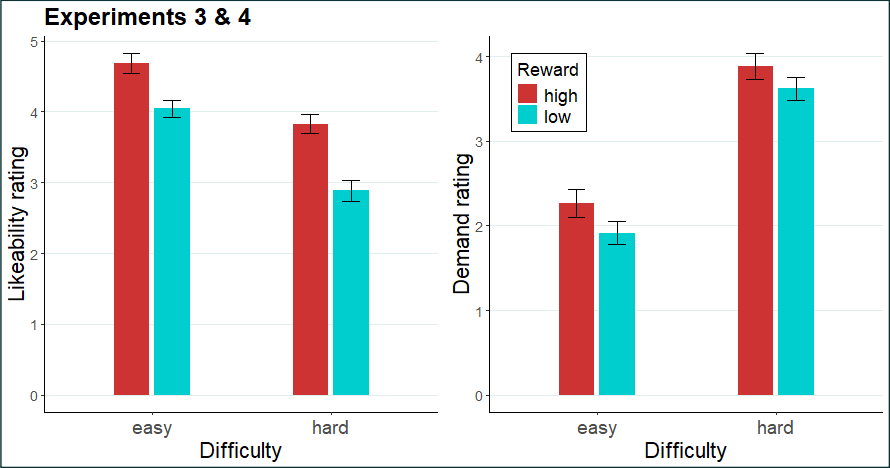
Likeability and demand ratings of evaluation cues across Experiments 3 and 4. *Note*. The likeability rating (left) and demand rating (right) of each e-cue across Experiment 3 and 4.

Similarly to Experiments 1 and 2, participants rated the likeability and demand of each of the e-cues (Figure 9). Again, participants rated the high reward cues (*M* = 4.26) to be more likeable than low reward cues (*M* = 3.46; *F*[1, 97] = 32.94, *p* < .001; collapsed across difficulty), and easy cues (*M* = 4.36) as more likeable than hard cues (*M* = 3.36; *F*[1, 97] = 40.39, *p* < .001; collapsed across reward). Additionally, a *reward* × *difficulty* interaction was significant in the cue likeability ratings (*F*[1, 97] = 3.96, *p* = .05), as likeability difference between high and low reward cues was larger in hard cues (*b =* 0.94, SE = 0.15, *t* = 6.09, *p* < .0001) compared to easy cues (*b =* 0.64, SE = 0.16, *t* = 4.04, *p* = .0001). High reward cues (*M* = 3.08) were rated as more demanding than low reward cues (*M* = 2.77; *F*[1, 97] = 9.76, *p* < .01), and hard cues (*M* = 3.76) as more demanding than easy cues (*M* = 2.09; *F*[1, 97] = 67.02, *p* < .001).

## Discussion

Using a cued RDM task (Experiments 1 and 2) and Stroop task (Experiments 3 and 4) across four experiments, we investigated whether people are more likely to regulate the amount of effort they invest in the task based on reward and difficulty cues when these cues have long versus short prediction horizons. In Experiments 1 and 3 cues had a short prediction horizon (i.e., reward and difficulty levels were cued for each trial), whereas Experiments 2 and 4 had a long prediction horizon (i.e., reward and difficulty levels were cued every six trials). In the long prediction horizon version of the RDM task, we observed a reward-dependent benefit in accuracy, especially in easy trials. In contrast, no reward effects were found with short prediction horizon cues. In the Stroop task a long prediction horizon induced a general reward benefit in reaction times that was not observed with short prediction horizon. These findings are consistent with the idea that there is a cost to cue-related effort regulation. When information pertaining to the optimal effort level changes often, in the case of a short prediction horizon, a better strategy may be to maintain a constant effort intensity rather than regulating effort based on each individual cue. In contrast, when information in the environment changes less often, the benefit of cue-related updating of effort levels outweighs the cost of the respective adjustments.

Despite the wide-spread understanding of effort as something that is adjusted dynamically with rapidly changing incentive conditions, our results suggest this might not always be the case. Models of effort have identified inherent costs of effort, like the opportunity cost (Otto & Daw, 2019) and the intensity cost (Musslick et al., 2015). However, these models have not taken into account the cost of effort regulation itself. Along with the findings reported here, a recent study by Grahek et al. (2024) provided support for this idea. Specifically, they manipulated the rate of change in cued reward information (high vs. low reward) in a colour Stroop task, and found that when reward information varies more, the reward-related differences in RTs and accuracies were larger in blocks where reward information was kept stable, compared to blocks where reward information varied across runs of six to nine trials. Their findings, similarly to ours, are consistent with the idea that there is a cost to regulating effort that can be amortised by a longer period of worthwhile effort. In their work Grahek and colleagues (2024) used the term “reconfiguration costs”, which they further formalized in the context of the expected value of control theory (Shenhav et al., 2013).

If the cost of adjusting effort is underlain by lingering effort configurations that constrain dynamic effort adaptations, if there was an endogenous “warm-up cost” to effort up-regulation, or if the miniblock cueing structure allows for a beneficial performance strategy, we would expect the effect of current reward condition to build up across the trials of a miniblock. We did not find evidence for such an explanation, insofar as the performance-boosting effect of reward was not modulated by the trial number in the easy condition in the RDM, or across difficulties in the Stroop task. However, it is possible that the sequential RDM stimulus presentation facilitated performance in Experiment 2. Nevertheless, in the Stroop task, we believe that cued effort information is used more when it pertains to more than a single one-off effort exertion, as part of a calculated strategy where the payoff of multiple successive trials offsets the cost of cue-related effort regulation.

Experiment 2, which involved the RDM with long prediction horizon cues, further revealed that the effect of reward information was most pronounced for low effort trials, which is indicative of efficient effort allocation based on expected costs and outcomes, paralleling previous observations in a visual discrimination task (Krebs et al., 2012). Similarly, Otto et al. (2022) found that in the context of task switching and a flanker task participants showed a performance benefit following reward cues, which over time was more pronounced in the easy condition compared to the hard condition. Otto et al. (2022) suggested this to be reflective of the marginal value of effort that is learnt over time. In other words, the cost of effort adjustment is higher when effort demands are high, and is consequentially less worth the effort. Conversely, when demands are low, effort adjustments require fewer resources and thus have lower inherent cost. Since in our visual discrimination task the effect of effort adjustments on performance might be mediated more by the perceptual properties of the task, the marginal value of effort could be learnt faster than in the more complex task-switching domain. The behavioural benefits of effort adjustments in the RDM are inherently more restricted by the perceptual properties of the stimuli, rendering the marginal value of effort in hard trials very low. This effect of marginal value of effort was not replicated in Stroop, possibly due to near ceiling accuracy even in the difficult trials of the two-response Stroop task.

The distinction between data- and resource-limited tasks (Norman & Bobrow, 1975), and how reward maximisation is achieved in each respectively, might explain why the reward effects emerged in accuracy in the RDM and in RT in the Stroop task. In the RDM, RT modulation is less feasible due to data-limited sensory evidence accumulation, suggested by the reward by difficulty interaction in accuracy and lack of it in RT. A satisfactory level of certainty can only be reached by accruing the needed perceptual evidence in each individual trial, allowing for less intra-individual variation in RT. Stroop performance on the other hand is less limited by the perceptual properties of the task, and is thus more dependent on the cognitive resources applied to the task, allowing for intentional, faster evidence accumulation without accuracy detriment. Fitting Stroop task performance with a drift diffusion model, the recent findings by Grahek and colleagues are in line with this (2024). They showed that high reward induced lower response threshold without changes in drift rate, indicating faster, but as effective, decision-making when incentivised. In contrast to the Stroop task, Bogacz and colleagues reported that in incentivised RDM the average thresholds were higher than required for reward optimisation (Bogacz et al., 2010). To conclude, in the Stroop task, but less so in the RDM, speeding up responding is a feasible strategy to ensure reward maximisation without compromising accuracy.

Despite replicating the interaction between reward and difficulty in the RDM and finding a main effect of reward in the Stroop task within our long prediction horizon manipulation, we did not find any reward-induced performance benefit in the RDM or Stroop task with a trial-wise cueing manipulation (Experiments 1 and 3). This contrasts with previous studies reporting performance boosting effects of reward using trial-by-trial cues in other task domains (e.g. Bundt et al., 2016; Padmala & Pessoa, 2011; Krebs et al., 2012), and in the Stroop task (Kostandyan et al., 2020). Possibly, this lack of a reward effect could be due to the fact that our cues signalled two types of information (about both reward and effort), leading to some form of goal neglect (e.g., Bhandari & Duncan, 2014; Brass et al., 2017). However, the overall lack of a main effect of reward in the RDM experiments could also be explained by it relying on data-limited processes more than the Stroop task. The parallel distributed processing model of the Stroop effect posits that Stroop task entails parallel activation of information about the word meaning and the ink colour (Cohen et al., 1990). In the case of incongruent trials this parallel activation results in cognitive conflict that can be resolved by enhancing the processing of the relevant stimulus property. The Stroop task is thus a more typical resource-limited task, and control-related areas like the dorsolateral prefrontal cortex and the anterior cingulate cortex have been shown to support Stroop conflict resolution (Botvinick et al., 2001). By contrast, dot-motion processing relies on a specialised visual area (V5; e.g. Britten et al., 1996), and microstimulation of this area speeds up RDM responses in monkeys (Ditterich et al., 2003). In humans, visual processing can be enhanced by directing visual attention like a spotlight (Posner et al., 1980). Due to global movement in the RDM stimuli, directing central attentional focus to the RDM target (i.e. coherently moving dots) does not aid target detection. In short, fast-paced effort regulation is likely to be less effective in the RDM than in the Stroop task due to RDM relying on sensory evidence accumulation.

The inclusion of intermediate difficulty trials in Experiments 1 and 2 enabled us to explore the additional question of whether differences in *expected difficulty* can lead to performance adjustments in trials of identical task difficulty. We hypothesised that expecting a demanding trial would be associated with performance benefits based on our previous observations of modulated neural activity in anticipation of a demanding target (Krebs et al. 2012; Schevernels et al. 2014). However, this hypothesis was not confirmed in the present data. Instead, we found some evidence for an opposite effect, in that when coupled with high reward, easy-cued trials were associated with higher accuracy than hard-cued across both experiments (although a marginally significant interaction suggested this effect to be stronger in Experiment 2). This suggests that participants did adjust their performance according to expected difficulty, especially when effort had additional motivational value. This finding, together with the observation that the reward benefit in regular trials emerged only in the easy trials, is consistent with the idea that participants are susceptible to the low marginal value of effort in the high difficulty condition. All the above being said, it is not possible to make strong inferences about the nature of difficulty expectation effects due to the small number of intermediate difficulty trials in these experiments. Difficulty cueing manipulations with a sufficiently large sample size and without the overshadowing effect of reward will be valuable to explore the motivational effects of difficulty expectations.

Effort allocation strategies have been shown to vary based on certain personality traits, such as the general affinity towards effort (as measured with the NFC scale) and reward responsiveness (as measured with the BIS-BAS reward responsiveness scale). For instance, individuals who like cognitive challenges more are less susceptible to external incentives (Sandra & Otto, 2018), suggesting that they consider effort investment as less costly. Other work has shown that high reward responsiveness predicts increased reward-related control adaptation (Braem et al., 2012). In the current study, we found that in the Stroop task, despite a lack of a main effect of reward in accuracy, high and intermediate levels of reward responsiveness were associated with higher accuracy in highly rewarded easy trials. This finding might reflect reward responsiveness modulating sensitivity to the marginal value of effort. Regarding affinity toward effort, high NFC predicted a reward effect in hard trials across the RDM experiments. In Experiment 4 higher NFC was associated with faster RT in high reward condition. This finding is at odds with past studies showing that high NFC is associated with no reward-facilitation in task switching (Sandra & Otto, 2018), and a higher subjective value of effort (Westbrook et al., 2013). It might be that the finding of Sandra and Otto (2018) reflected high NFC taking longer to disengage from a task, leading to larger switch cost. No NFC moderated reward effects were observed in the Stroop experiment with short prediction horizon, which might be evidence of high NFC relating to the tendency to form stronger control signals (Musslick et al., 2019), maybe in part facilitating cue use with long prediction horizon.

Across four experiments and two task domains, we contrasted reward and difficulty cues with short versus long prediction horizons, and demonstrated that reward motivation was highest after cues with a long prediction horizon. We found no significant effect of miniblock trial number, suggesting that cues influenced performance throughout a miniblock in both tasks. However, it is possible that in the more data-limited RDM the stronger effort adjustments with long cue prediction horizon were a mix of performance facilitation by sequential presentation of RDM stimuli as well as evaluation processes. In conclusion, in Stroop and to an extent in RDM, it is likely that the cost of effort adjustment arises from a value-based decision, rather than bottom-up constraints of effort allocation itself.

## Data Accessibility Statement

Data is available at https://osf.io/d6ber/.

## Additional File

The additional file for this article can be found as follows:

10.5334/joc.415.s1 Supplementary Materials. Tables S1–S14.

## Footnotes

Due to a technical issue in Experiment 1, the random-dot-motion stimuli were shifted to the left of the fixation cross for two participants. Excluding those participants from the analyses did not change the results. We therefore decided to keep them in the final sample.

In our earlier unpublished study, 30% coherence yielded performance approximately halfway between the RTs and accuracy associated with easy and hard trials. Suggestive of a non-linear effect of coherence on performance, 35% coherence produced performance closer to that in easy trials (50% coherence).

\[Adjusted\ accuracy\left(\% \right)\; = \;80\, + \;\left({true\ accuracy\; - \;80} \right)\; \times \;.75\; + \;x\; - \;2\] *x* = random number between 0 and 4.
