# Supplementary material for: The Cost of Regulating Effort: Reward and Difficulty Cues With Longer Prediction Horizons Have a Stronger Impact on Performance
Source: J Cogn. 2025 Jan 7;8(1):9. doi: 10.5334/joc.415 (PMC11720864; doi:10.5334/joc.415)
Supplement: Supplementary Materials. — Tables S1–S14. [file joc-8-1-415-s1.pdf]

Supplementary materials

Table S1. Output of a GLMM of accuracy in Experiment 2 with random effect structure *difficulty | participant*

	Accuracy		
Predictors	*Odds Ratios*	*CI*	*p*
(Intercept)	6.36	5.04 – 8.03	**<0.001**
Reward (high)	1.08	1.02 – 1.15	**0.014**
Difficulty (easy)	2.13	1.88 – 2.42	**<0.001**
Reward (high) * Difficulty (easy)	1.08	1.02 – 1.15	**0.010**
**Random Effects**			
σ^2	3.29		
τ_{00} participant	0.62		
τ_{11} participant.Difficulty (easy)	0.12		
ρ_{01} participant	0.81		
ICC	0.18		
N participant	49		
Observations	9092		
Marginal R^2 / Conditional R^2	0.127 / 0.287		

Table S2. Output of a GLMM of accuracy in Experiment 1 with random effect structure *difficulty | participant*

	Accuracy		
Predictors	*Odds Ratios*	*CI*	*p*
(Intercept)	7.32	5.25 – 10.20	**<0.001**
Reward (High)	0.99	0.93 – 1.05	0.628
Difficulty (easy)	2.11	1.81 – 2.46	**<0.001**
Reward (High) * Difficulty (easy)	0.98	0.93 – 1.05	0.593
**Random Effects**			
σ^2	3.29		
τ_{00} participant	1.19		

τ_{11} participant.Difficulty (high)	0.17
ρ_{01} participant	0.97
ICC	0.29
N participant	45
Observations	8958
Marginal R^2 / Conditional R^2	0.107 / 0.368

Table S3. Output of a GLMM of accuracy in Experiment 2 with predictor variable trial with random effect structure *difficulty | participant*

	**Accuracy**		
Predictors	*Odds Ratios*	*CI*	*p*
(Intercept)	6.42	5.08 – 8.11	**<0.001**
Reward (high)	1.08	1.01 – 1.15	**0.015**
Difficulty (easy)	2.14	1.89 – 2.43	**<0.001**
Difficulty (intermediate)	0.94	0.84 – 1.04	0.221
trial [1]	1.05	0.99 – 1.12	0.127
trial [2]	0.95	0.91 – 0.99	**0.012**
trial [3]	1.00	0.97 – 1.04	0.827
trial [4]	1.00	0.97 – 1.02	0.797
trial [5]	6.42	5.08 – 8.11	**<0.001**
Reward (high) * Difficulty (easy)	1.08	1.02 – 1.15	**0.011**
Reward (high) * trial [1]	1.02	0.91 – 1.13	0.784
Reward (high) * trial [2]	1.03	0.97 – 1.10	0.301
Reward (high) * trial [3]	1.02	0.98 – 1.07	0.303
Reward (high) * trial [4]	1.00	0.97 – 1.04	0.792
Reward (high) * trial [5]	1.00	0.97 – 1.02	0.830
Difficulty (easy) * trial [1]	1.04	0.94 – 1.16	0.424
Difficulty (easy) * trial [2]	0.99	0.93 – 1.06	0.789
Difficulty (easy) * trial [3]	0.96	0.92 – 1.00	0.056

Difficulty (easy) * trial [4]	0.99	0.95 – 1.02	0.378
Difficulty (easy) * trial [5]	1.02	0.99 – 1.05	0.159
(Reward (high) * Difficulty (easy)) * trial [1]	1.07	0.96 – 1.19	0.210
(Reward (high) * Difficulty (easy)) * trial [2]	1.06	0.99 – 1.13	0.076
(Reward (high) * Difficulty (easy)) * trial [3]	1.00	0.96 – 1.04	0.995
(Reward (high) * Difficulty (easy)) * trial [4]	1.00	0.97 – 1.04	0.777
(Reward (high) * Difficulty (easy)) * trial [5]	1.00	0.98 – 1.03	0.752
**Random Effects**			
σ^2	3.29		
$\tau_{00\ participant}$	0.62		
$\tau_{11\ participant.Difficulty\ (easy)}$	0.13		
$\rho_{01\ participant}$	0.81		
ICC	0.18		
$N_{participant}$	49		
Observations	9092		
Marginal R^2 / Conditional R^2	0.133 / 0.293		

Table S4. Output of a GLMM of RT across Experiment 1 and Experiment 2 including the predictor variable reward responsiveness with random effect structure *reward * difficulty | participant*

	RT		
Predictors	*Estimates*	*CI*	*p*
(Intercept)	545.42	513.12 – 579.75	**<0.001**
Reward (high)	1.00	0.99 – 1.00	0.148
Difficulty (easy)	0.95	0.94 – 0.96	**<0.001**
Experiment 2	1.02	0.96 – 1.08	0.629
Reward Resp.	0.99	0.93 – 1.05	0.701

Reward (high) * Difficulty (easy)	1.00	1.00 – 1.01	0.214
Reward (high) * Experiment 2	1.00	0.99 – 1.00	0.459
Difficulty (easy) * Experiment 2	1.00	0.99 – 1.01	0.523
Reward (high) * Reward Resp.	1.00	0.99 – 1.00	0.625
Difficulty (easy) * Reward Resp.	1.00	0.99 – 1.01	0.965
Experiment 2 * Reward Resp.	0.95	0.89 – 1.01	0.125
Reward (high) * Difficulty (easy) * Experiment 2	1.00	0.99 – 1.00	0.755
(Reward (high) * Difficulty (easy)) * Reward Resp.	1.00	1.00 – 1.01	0.128
(Reward (high) * Experiment 2) * Reward Resp.	1.00	1.00 – 1.01	0.071
(Difficulty (easy) * Experiment 2) * Reward Resp.	1.01	0.99 – 1.02	0.370
(Reward (high) * Difficulty (easy) * Experiment 2) * Reward Resp.	1.00	1.00 – 1.01	0.667
**Random Effects**			
σ^2	0.05		
$\tau_{00\ \text{participant}}$	0.01		
$\tau_{11\ \text{participant.Reward (high)}}$	0.00		
$\tau_{11\ \text{participant.Difficulty (easy)}}$	0.03		
$\tau_{11\ \text{participant. Reward (high):Difficulty (easy)}}$	0.01		
ρ_{01}	0.03		
	-0.62		
	-0.04		
ICC	0.24		
$N_{\text{participant}}$	94		
Observations	14780		
Marginal R^2 / Conditional R^2	0.077 / 0.296		

Table S5. Output of a GLMM of accuracy across Experiment 1 and Experiment 2 including the predictor variable reward responsiveness with random effect structure *difficulty | participant*

	Accuracy		
Predictors	*Odds Ratios*	*CI*	*p*
(Intercept)	6.82	5.60 – 8.32	**<0.001**
Reward (high)	1.03	0.99 – 1.08	0.154
Difficulty (easy)	2.14	1.94 – 2.35	**<0.001**
Experiment 2	0.95	0.78 – 1.16	0.602
Reward Resp.	0.93	0.76 – 1.13	0.443
Reward (high) * Difficulty (easy)	1.03	0.99 – 1.08	0.142
Reward (high) * Experiment 2	1.05	1.00 – 1.09	**0.042**
Difficulty (easy) * Experiment 2	1.02	0.93 – 1.11	0.734
Reward (high) * Reward Resp.	0.99	0.95 – 1.03	0.631
Difficulty (easy) * Reward Resp.	1.03	0.94 – 1.13	0.573
Experiment 2 * Reward Resp.	0.97	0.80 – 1.19	0.797
Reward (high) * Difficulty (easy) * Experiment 2	1.05	1.01 – 1.10	**0.029**
(Reward (high) * Difficulty (easy)) * Reward Resp.	1.02	0.97 – 1.07	0.427
(Reward (high) * Experiment 2) * Reward Resp.	1.01	0.97 – 1.06	0.631
(Difficulty (easy) * Experiment 2) * Reward Resp.	0.91	0.83 – 1.00	0.056
(Reward (high) * Difficulty (easy) * Experiment 2) * Reward Resp.	0.97	0.93 – 1.02	0.267
**Random Effects**			
σ^2	3.29		
$\tau_{00\ participant}$	0.86		
$\tau_{11\ participant.Difficulty\ (easy)}$	0.14		
$\rho_{01\ participant}$	0.93		
ICC	0.23		
$N_{participant}$	94		
Observations	18050		
Marginal R^2 / Conditional R^2	0.121 / 0.326		

Table S6. Output of a GLMM of RT across Experiment 1 and Experiment 2 including the predictor variable NFC with random effect structure *1 | participant*

		**RT**	
Predictors	*Estimates*	*CI*	*p*
(Intercept)	542.34	510.39 – 576.29	**<0.001**
Reward (high)	1.00	0.99 – 1.00	0.214
Difficulty (easy)	0.95	0.94 – 0.96	**<0.001**
Experiment 2	1.01	0.95 – 1.08	0.652
NFC	1.02	0.96 – 1.09	0.504
Reward (high) * Difficulty (easy)	1.00	1.00 – 1.01	0.196
Reward (high) * Experiment 2	1.00	0.99 – 1.00	0.442
Difficulty (easy) * Experiment 2	1.00	0.99 – 1.01	0.528
Reward (high) * NFC	1.00	0.99 – 1.00	0.418
Difficulty (easy) * NFC	1.00	0.99 – 1.01	0.798
Experiment 2 * NFC	0.95	0.90 – 1.01	0.135
Reward (high) * Difficulty (easy) * Experiment 2	1.00	0.99 – 1.01	0.887
(Reward (high) * Difficulty (easy)) * NFC	1.01	1.00 – 1.01	**0.045**
(Reward (high) * Experiment 2) * NFC	1.00	1.00 – 1.01	0.740
(Difficulty (easy) * Experiment 2) * NFC	1.00	0.99 – 1.01	0.869
(Reward (high) * Difficulty (easy) * Experiment 2) * NFC	1.00	0.99 – 1.00	0.405

Random Effects

σ^2	0.05
τ_{00} participant	0.01
τ_{11} participant.Reward (high)	0.00
τ_{11} participant.Difficulty (easy)	0.00
τ_{11} participant. Reward (high):Difficulty (easy)	0.00
ρ_{01}	-0.09
	-0.06
	0.00
ICC	0.16
N random_ID	94
Observations	14780
Marginal R^2 / Conditional R^2	0.088 / 0.236

Table S7. Output of a GLMM of accuracy across Experiment 1 and Experiment 2 including the predictor variable NFC with random effect structure *difficulty | participant*

	Accuracy		
Predictors	*Odds Ratios*	*CI*	*p*
(Intercept)	6.79	5.58 – 8.26	**<0.001**
Difficulty (easy)	1.03	0.99 – 1.08	0.147
Reward (high)	2.11	1.92 – 2.33	**<0.001**
Experiment 2	0.95	0.78 – 1.15	0.579
NFC	1.03	0.85 – 1.25	0.787
Difficulty (easy) * Reward (high)	1.03	0.99 – 1.08	0.131
Difficulty (easy) * Experiment 2	1.05	1.00 – 1.09	**0.037**
Reward (high) * Experiment 2	1.02	0.93 – 1.11	0.718
Difficulty (easy) * NFC	1.01	0.97 – 1.06	0.563
Reward (high) * NFC	0.98	0.90 – 1.08	0.738
Experiment 2 * NFC	1.07	0.88 – 1.30	0.524

Difficulty (easy) * Reward (high) * Experiment 2	1.05	1.01 – 1.10	**0.027**
(Difficulty (easy) * reward cue1) * NFC	1.01	0.97 – 1.06	0.622
(Difficulty (easy) * Experiment 2) * NFC	1.02	0.97 – 1.06	0.422
(Reward (high) * Experiment 2) * NFC	0.99	0.90 – 1.09	0.848
(Difficulty (easy) * Reward (high) * Experiment 2) * NFC	1.02	0.98 – 1.07	0.336
**Random Effects**			
σ^2	3.29		
τ_{00} participant	0.85		
τ_{11} participant.Difficulty (easy)	0.14		
ρ_{01}	0.91		
ICC	0.23		
N participant	94		
Observations	18050		
Marginal R^2 / Conditional R^2	0.119 / 0.323		

Table S8. Output of a GLMM of RT in Experiment 4 with random effect structure *reward * difficulty | participant*

	RT		
Predictors	*Estimates*	*CI*	*p*
(Intercept)	401.14	385.44 – 417.47	**<0.001**
Reward (high)	0.99	0.99 – 1.00	**0.011**
Difficulty (easy)	0.99	0.98 – 1.00	0.073
Reward (high) * Difficulty (easy)	1.00	1.00 – 1.01	0.600
**Random Effects**			
σ^2	0.03		
τ_{00} participant	0.00		
τ_{11} participant.Reward (high)	0.00		

τ_{11} participant.Difficulty (easy)	0.00
τ_{11} participant.Reward (high):Difficulty (easy)	0.00
ρ_{01}	0.05
	-0.15
	-0.09
ICC	0.07
N participant	48
Observations	12718
Marginal R^2 / Conditional R^2	0.003 / 0.072

Table S9. Output of a GLMM of RT in Experiment 3 with random effect structure *reward * difficulty | participant*

	RT		
Predictors	*Estimates*	*CI*	*p*
(Intercept)	408.30	389.95 – 427.50	**<0.001**
Reward (high)	1.00	0.99 – 1.00	0.349
Difficulty (easy)	0.99	0.99 – 1.00	0.079
Reward (high) * Difficulty (easy)	1.00	1.00 – 1.01	0.879
**Random Effects**			
σ^2	0.03		
τ_{00} participant	0.00		
τ_{11} participant.Reward (high)	0.00		
τ_{11} participant.Difficulty (easy)	0.00		
τ_{11} participant.Reward (high):Difficulty (easy)	0.00		
ρ_{01}	-0.09		
	-0.10		
	0.11		
ICC	0.08		
N participant	50		
Observations	12829		
Marginal R^2 / Conditional R^2	0.001 / 0.081		

Table S10. Output of a GLMM of RT in Experiment 4 including the predictor variable *trial* with random effect structure *1 | participant*

	RT		
Predictors	*Estimates*	*CI*	*p*
(Intercept)	401.97	386.20 – 418.39	**<0.001**
Reward (high)	0.99	0.99 – 1.00	**<0.001**
Difficulty (easy)	0.99	0.99 – 0.99	**<0.001**
trial [1]	0.96	0.96 – 0.97	**<0.001**
trial [2]	0.98	0.98 – 0.99	**<0.001**
trial [3]	0.99	0.99 – 0.99	**<0.001**
trial [4]	0.99	0.99 – 0.99	**<0.001**
trial [5]	1.00	1.00 – 1.00	**<0.001**
Reward (high) * Difficulty (easy)	1.00	1.00 – 1.00	0.261
Reward (high) * trial [1]	1.00	1.00 – 1.01	0.821
Reward (high) * trial [2]	1.00	1.00 – 1.01	0.061
Reward (high) * trial [3]	1.00	1.00 – 1.00	0.595
Reward (high) * trial [4]	1.00	1.00 – 1.00	0.118
Reward (high) * trial [5]	1.00	1.00 – 1.00	0.707
Difficulty (easy) * trial [1]	1.00	0.99 – 1.00	0.358
Difficulty (easy) * trial [2]	1.00	1.00 – 1.00	0.164
Difficulty (easy) * trial [3]	1.00	1.00 – 1.00	0.735
Difficulty (easy) * trial [4]	1.00	1.00 – 1.00	0.350
Difficulty (easy) * trial	1.00	1.00 – 1.00	0.444

[5]			
(Reward (high) * Difficulty (easy)) * trial [1]	1.00	0.99 – 1.00	0.838
(Reward (high) * Difficulty (easy)) * trial [2]	1.00	1.00 – 1.00	0.169
(Reward (high) * Difficulty (easy)) * trial [3]	1.00	1.00 – 1.00	0.750
(Reward (high) * Difficulty (easy)) * trial [4]	1.00	1.00 – 1.00	0.957
(Reward (high) * Difficulty (easy)) * trial [5]	1.00	1.00 – 1.00	0.828
**Random Effects**			
σ^2	0.03		
$\tau_{00\ participant}$	0.00		
ICC	0.06		
N participant	48		
Observations	12718		
Marginal R^2 / Conditional R^2	0.041 / 0.096		

Table S11. Output of a GLMM of RT across Experiment 3 and Experiment 4 including the predictor variable reward responsiveness with random effect structure *1 | participant*

	RT		
Predictors	*Estimates*	*CI*	*p*
(Intercept)	406.27	393.98 – 418.95	**<0.001**
Reward (high)	1.00	0.99 – 1.00	**<0.001**
Difficulty (easy)	0.99	0.99 – 0.99	**<0.001**
Experiment 3	0.99	0.96 – 1.02	0.550

Reward Resp.	0.99	0.96 – 1.03	0.702
Reward (high) * Difficulty (easy)	1.00	1.00 – 1.00	0.540
Reward (high) * Experiment 4	1.00	1.00 – 1.00	**0.022**
Difficulty (easy) * Experiment 4	1.00	1.00 – 1.00	0.139
Reward (high) * Reward Resp.	1.00	1.00 – 1.00	0.104
Difficulty (easy) * Reward Resp.	1.00	1.00 – 1.00	**0.030**
Experiment 3 * Reward Resp.	1.02	0.99 – 1.05	0.300
Reward (high) * Difficulty (easy) * Experiment 4	1.00	1.00 – 1.00	0.473
(Reward (high) * Difficulty (easy)) * Reward Resp.	1.00	1.00 – 1.00	0.691
(Reward (high) * Experiment 4) * Reward Resp.	1.00	1.00 – 1.00	**0.042**
(Difficulty (easy) * Experiment 4) * Reward Resp.	1.00	1.00 – 1.00	0.414
(Reward (high) * Difficulty (easy) * Experiment 4) * Reward Resp.	1.00	1.00 – 1.00	**0.044**
**Random Effects**			
σ^2	0.03		
$\tau_{00\ participant}$	0.00		
ICC	0.06		
$N_{participant}$	98		
Observations	25547		
Marginal R^2 /	0.015 / 0.077		

Conditional R^2

Table S12. Output of a GLMM of accuracy across Experiment 3 and Experiment 4 including the predictor variable reward responsiveness with random effect structure *difficulty | participant*

	Accuracy		
Predictors	*Odds Ratios*	*CI*	*p*
(Intercept)	20.28	17.18 – 23.94	**<0.001**
Difficulty (easy)	1.17	1.09 – 1.26	**<0.001**
Reward (high)	1.05	0.99 – 1.10	0.098
Experiment 4	1.20	1.02 – 1.42	**0.026**
Reward Resp.	0.98	0.83 – 1.16	0.848
Difficulty (easy) * Reward (high)	1.04	0.98 – 1.09	0.192
Difficulty (easy) * Experiment 4	1.03	0.97 – 1.10	0.306
Reward (high) * Experiment 4	1.02	0.96 – 1.07	0.545
Difficulty (easy) * Reward Resp.	1.05	0.98 – 1.11	0.161
Reward (high) * Reward Resp.	1.00	0.94 – 1.05	0.864
Experiment 4 * Reward Resp.	1.00	0.84 – 1.17	0.960
(Difficulty (easy) * reward cue1) * Experiment 4	1.01	0.96 – 1.06	0.780
(Difficulty (easy) * Reward (high)) * Reward Resp.	1.06	1.01 – 1.12	**0.031**
(Difficulty (easy) * Experiment 4) * Reward Resp.	0.98	0.92 – 1.04	0.446
(Reward (high) * Experiment 4) * Reward Resp.	1.02	0.96 – 1.07	0.525
(Difficulty (easy) * Reward (high) * Experiment 4) * Reward Resp.	1.05	0.99 – 1.10	0.101
**Random Effects**			
σ^2	3.29		
$\tau_{00\ \text{participant}}$	0.58		

τ_{11} participant.Difficulty (easy)	0.02
ρ_{01} participant	-0.78
ICC	0.15
N participant	98
Observations	27233
Marginal R^2 / Conditional R^2	0.019 / 0.170

Table S13. Output of a GLMM of RT across Experiment 3 and Experiment 4 including the predictor variable NFC with random effect structure *1 | participant*

	RT		
Predictors	*Estimates*	*CI*	*p*
(Intercept)	405.25	393.00 – 417.89	**<0.001**
Reward (high)	1.00	0.99 – 1.00	**<0.001**
Difficulty (easy)	0.99	0.99 – 0.99	**<0.001**
Experiment 4	0.99	0.96 – 1.02	0.582
NFC	1.00	0.97 – 1.03	0.815
Reward (high) * difficulty cue1	1.00	1.00 – 1.00	0.315
Reward (high) * Experiment 4	1.00	1.00 – 1.00	**0.032**
Difficulty (easy) * Experiment 4	1.00	1.00 – 1.00	0.239
Reward (high) * NFC	1.00	1.00 – 1.00	0.064
Difficulty (easy) * NFC	1.00	1.00 – 1.00	0.534
Experiment 4 * NFC	1.00	0.97 – 1.03	0.983
Reward (high) * Difficulty (easy) * Experiment 4	1.00	1.00 – 1.00	0.525
(Reward (high) * Difficulty (easy)) * NFC	1.00	1.00 – 1.00	0.710
(Reward (high) * Experiment 4) * NFC	1.00	1.00 – 1.00	**0.034**

(Difficulty (easy) * Experiment 4) * NFC	1.00	1.00 – 1.00	0.442
(Reward (high) * Difficulty (easy) * Experiment 4) * NFC	1.00	1.00 – 1.00	0.125
**Random Effects**			
σ^2	0.03		
$\tau_{00\ participant}$	0.00		
ICC	0.06		
$N_{participant}$	98		
Observations	25547		
Marginal R^2 / Conditional R^2	0.006 / 0.069		

Table S14. Output of a GLMM of accuracy across Experiment 3 and Experiment 4 including the predictor variable NFC with random effect structure *difficulty | participant*

		**Accuracy**	
Predictors	*Odds Ratios*	*CI*	*p*
(Intercept)	20.32	17.25 – 23.93	**<0.001**
Reward (high)	1.05	0.99 – 1.10	0.083
Difficulty (easy)	1.17	1.10 – 1.25	**<0.001**
Experiment 4	1.20	1.02 – 1.41	**0.026**
NFC	0.96	0.81 – 1.13	0.608
Reward (high) * difficulty cue1	1.03	0.97 – 1.08	0.326
Reward (high) * Experiment 4	1.02	0.96 – 1.07	0.559
Difficulty (easy) * Experiment 4	1.03	0.97 – 1.09	0.376
Reward (high) * NFC	1.01	0.95 – 1.06	0.837
Difficulty (easy) * NFC	1.01	0.95 – 1.07	0.750
Experiment 4 * NFC	1.04	0.88 – 1.23	0.619
Reward (high) * Difficulty (easy) * Experiment 4	1.00	0.95 – 1.06	0.941

(Reward (high) * Difficulty (easy)) * NFC	1.00	0.95 – 1.06	0.930
(Reward (high) * Experiment 4) * NFC	1.04	0.99 – 1.10	0.125
(Difficulty (easy) * Experiment 4) * NFC	0.94	0.88 – 1.00	**0.041**
(Reward (high) * Difficulty (easy) * Experiment 4) * NFC	0.97	0.92 – 1.02	0.200
**Random Effects**			
σ^2	3.29		
$\tau_{00\ \text{participant}}$	0.58		
$\tau_{11\ \text{participant.Difficulty (easy)}}$	0.02		
$\rho_{01\ \text{participant}}$	-0.80		
ICC	0.15		
$N_{\text{participant}}$	98		
Observations	27233		
Marginal R^2 / Conditional R^2	0.019 / 0.169		
